# Clinical Manifestation and Obstetric Outcomes in Pregnant Women with SARS-CoV-2 Infection at Delivery: A Retrospective Cohort Analysis

**DOI:** 10.3390/jpm12091480

**Published:** 2022-09-09

**Authors:** Gordana Grgić, Anis Cerovac, Igor Hudić, Antonio Simone Laganà, Alessandro Favilli, Simone Garzon, Vito Chiantera, Chrysoula Margioula-Siarkou, Azra Hadžimehmedović, Amer Mandžić

**Affiliations:** 1 Clinic for Gynaecology and Obstetrics, University Clinical Centre Tuzla, 75000 Tuzla, Bosnia and Herzegovina; 2 School of Medicine, University of Tuzla, 75000 Tuzla, Bosnia and Herzegovina; 3 Department of Gynaecology and Obstetrics, General Hospital Tešanj, 74260 Tešanj, Bosnia and Herzegovina; 4 Unit of Gynecologic Oncology, ARNAS “Civico-Di Cristina-Benfratelli”, Department of Health Promotion, Mother and Child Care, Internal Medicine and Medical Specialties (PROMISE), University of Palermo, 90127 Palermo, Italy; 5 Department of Obstetrics and Gynecology, AOUI Verona, University of Verona, 37126 Verona, Italy; 6 2nd Academic Department of Obstetrics and Gynaecology, Hippokration General Hospital, Aristotle University of Thessaloniki, 546 42 Thessaloníki, Greece

**Keywords:** clinical manifestation, obstetric outcomes, maternal-neonatal outcome, pregnancy, SARS-CoV-2, COVID-19

## Abstract

This retrospective cohort study aimed to analyze the clinical manifestations, complications, and maternal-fetal outcomes in patients affected by severe acute respiratory syndrome coronavirus 2 (SARS-CoV-2) infection during delivery. The cohort included 61 pregnant women positive for SARS-CoV-2 infection at the time of delivery. Patients were divided into two groups: symptomatic and asymptomatic. We found a significantly higher rate of leukocytosis (*p* < 0.00078) and lymphopenia (*p* < 0.0024) in symptomatic women compared with asymptomatic ones. Other laboratory parameters, such as CRP (*p* = 0.002), AST (*p* = 0.007), LDH (*p* = 0.0142), ferritin (*p* = 0.0036), and D-dimer (*p* = 0.00124), were also significantly more often increased in the group of symptomatic pregnant women. Overall, symptomatic pregnant women with SARS-CoV-2 infection at the delivery show more often altered laboratory parameters compared with asymptomatic ones; nevertheless, they have a slightly higher but non-significant rate of preterm delivery, cesarean section, as well as lower neonatal birth weight and Apgar score, compared with asymptomatic women.

## 1. Introduction

Coronavirus disease 2019 (COVID-19) is a syndrome that often involves a respiratory infection caused by severe acute respiratory syndrome coronavirus 2 (SARS-CoV-2) virus [1]. The clinical manifestations of COVID-19 are similar to the severe acute respiratory syndrome (SARS) and Middle East respiratory syndrome (MERS) infections [2]. Hospitalized patients often develop severe pneumonia, 17–29% of the cases develop acute respiratory distress syndrome, and 23–32% require admission to intensive care units [3]. The most common symptoms are fever (83–100%), cough (59–82%), muscle pain (11–35%), headache (7–8%), and diarrhea (2–10%); in addition, COVID-19 infection is often associated with alterations of the chest X-ray [3].

The risk of developing SARS-CoV-2 infection in pregnant women is the same as in the general population [4]. However, physiological changes in pregnancy, such as a decrease in pulmonary residual functional volume and edema of the respiratory tract’s mucous membrane, as well as immune changes, can increase susceptibility to viral infections and lead to more serious consequences [5,6,7]. Infections with SARS and MERS viruses have shown increased morbidity in pregnant women and fetuses [8], so pregnant women belong to a high-risk group [8,9,10].

An analysis of 42 studies involving 438.548 pregnant women, comparing the ones with and without SARS-CoV-2 infection, found that pregnant women with SARS-CoV-2 infection were more likely to have preeclampsia, preterm birth, and increased perinatal mortality, as well as neonates with low birth weight [11]. This may be due, at least in part, to physiological changes during pregnancy, which include cardiovascular, respiratory, and immune systems, which can also result in a different response to infection. Some studies have also suggested an increased risk of fetal complications [12]. The increased risk of fetal complications could be due, at least in part, to altered placental functions: indeed, a recent analysis about placental histomorphology and pregnancy outcomes showed that decidual vasculopathy, maternal vascular thrombosis, and chronic histiocytic intervillositis were significantly higher in the COVID-19-infected placentas when compared with placentas from non-infected healthy pregnancies [13]: this is likely to be, at least in part, the underlying cause of preterm delivery, low neonatal birth weight, and low Apgar score in pregnant women affected by COVID-19 infection.

Available data on maternal infection include several risks for the fetus, such as fetal distress, abortion, premature birth, neonatal low birth weight, and stillbirth [8]. Available studies have shown that the vertical transmission of the virus is possible and that this is mostly a placental transfer of the virus [14]. Information about the presence of the virus in the female genital tract, which could be important for vertical and sexual transmission of the virus, is limited. In addition, the optimal delivery mode for infected pregnant women needs to be based on obstetric indications and COVID-19 severity [15,16,17].

In this retrospective cohort study, we aimed to analyze the clinical manifestations, complications, and maternal–fetal outcomes of patients with proven SARS-CoV-2 infection during delivery, comparing symptomatic and asymptomatic women in Bosnia and Herzegovina. A previous study included SARS-CoV-2-infected pregnant women in any trimester of pregnancy in this setting [18], whereas in the present study we focused specifically on SARS-CoV-2-infected pregnant women at delivery.

## 2. Materials and Methods

We retrospectively reviewed the prospectively collected delivery room database regarding the period from 15 March 2020 to 31 October 2021, after receiving a formal Institutional Review Board approval (ID: 02-09/2-77/21). The design, analysis, interpretation of data, drafting, and revisions conform with the Helsinki Declaration, the Committee on Publication Ethics guidelines, and the Strengthening the Reporting of Observational Studies in Epidemiology Statement [19], available through the Enhancing the Quality and Transparency of Health Research Network. The data collected were anonymized, taking into account the observational nature of the study, without personal data that could lead to the formal identification of the patient. Each patient enrolled in this study signed a consent form to allow data collection and analysis for research purposes. The study was not advertised. No remuneration was offered to the patients to provide their consent.

During the study period, there were a total of 4081 deliveries. Since the declaration of the COVID-19 pandemic, all hospitalized patients have undergone a PCR test for SARS-CoV-2 infection. SARS-CoV-2 infected patients were then allocated to a separate ward and delivery room, where they were managed according to international guidelines and recommendations [20,21]. We identified from the delivery room database and included patients with a positive SARS-CoV-2 PCR test at the time of delivery, subdividing them into two groups: symptomatic and asymptomatic. The following clinical characteristics were retrieved from the prospectively collected database: age, parity, associated diseases in pregnancy, and the presence of pneumonia confirmed by X-ray of the lungs. Among the laboratory parameters, we retrieved and analyzed data about blood count, C reactive protein (CRP), aspartate aminotransferase (AST), alanine aminotransferase (ALT), lactate dehydrogenase (LDH), ferritin, D-dimer value, and creatine kinase (CK). Analyzed data about the condition of the newborn at birth were the Apgar scores after the first and fifth minute, neonatal weight (low weight was defined as <2499 gr), the gestational week at the time of birth (preterm delivery was defined when occurring before 37 gestational weeks), and the type of delivery (vaginal delivery or cesarean section). Any data missing in the prospectively collected database were extracted from medical records.

Statistical Analysis

Statistical analysis was performed with InStat 3.10, GraphPad Software, San Diego, CA, USA. The Shapiro–Wilk test was used to check whether data were normally distributed. Continuous variables were expressed as mean and standard deviation (SD) or median and interquartile range (IQR), as appropriate. Categorical variables were expressed as frequency and percentage. The independent t-test and Wilcoxon rank-sum test were used to compare continuous variables, as appropriate. The χ^2^ test and Fisher’s exact test were used to compare categorical data. A *p* value < 0.05 was considered statistically significant.

## 3. Results

We included 61 pregnant women: 32 patients had symptoms, whereas 29 patients were asymptomatic. Among 32 pregnant women who had symptoms, the presence of pneumonia was found using X-ray in 90.6% of the cases, while in the group of asymptomatic pregnant women, pneumonia was not diagnosed regardless of a positive SARS-CoV-2 PCR test. Among the symptomatic women, the most common symptoms were cough and fever. These two symptoms were found in 20 patients (62.5%). Five (15.6%) pregnant women had more severe symptoms, such as difficulty breathing and choking cough. Among other symptoms, one (3.12%) pregnant woman had a severe headache, and another one (3.12%) had vomiting.

As shown in Table 1, the two groups did not differ significantly for baseline parameters such as age (*p* = 0.71) or parity (*p* = 0.305), or for the rate of other comorbidities.

Clinical and laboratory data are reported in Table 2. Among symptomatic women, 29 (90.6%) developed pneumonia. Symptomatic women had a higher rate of leukocytosis (*p* < 0.00078) and lymphopenia (*p* < 0.0024) than asymptomatic patients. Other laboratory parameters, such as CRP (*p* = 0.002), AST (*p* = 0.007), LDH (*p* = 0.0142), ferritin (*p* = 0.0036), and D-dimer (*p* = 0.00124), were also statistically significantly higher in the group of symptomatic pregnant women.

Symptomatic pregnant women were more often treated with antibiotics (*p* = 0.024) or antibiotics plus low-molecular-weight heparin (*p* = 0.00328). Only in the group of symptomatic patients, we observed pregnant women treated with corticosteroids (*p* = 0.17), oxygen (*p* = 0.026), or women admitted to the intensive care unit (*p* = 0.17) (Table 3). The only two pregnant women that were transferred to the intensive care unit due to the worsening of their overall condition had a favorable outcome, after intensive treatment. No fatalities have been reported in in our cohort.

As shown in Table 4, we found a slightly higher—but non-significant—rate of preterm delivery (*p* = 0.673), cesarean section (*p* = 0.185), neonatal with low birth weight (*p* = 0.201), and Apgar score ≤ 7 at the first minute (*p* = 0.519) in symptomatic pregnant women compared with asymptomatic ones.

## 4. Discussion

Previous data on SARS and MERS infection during pregnancy have shown that it may be an asymptomatic case leading to severe disease and death. The most common symptoms of COVID-19 infection are fever and cough, which over 80% of hospitalized patients complain about [22]. As confirmed by a recent meta-analysis, in pregnant women the most common symptoms are also fever (40%) and cough (39%) [23]. Interestingly, in our series we found pneumonia at chest X-ray in almost all (90.6%) symptomatic pregnant women, whereas none of the asymptomatic pregnant women developed radiological signs of pneumonia at chest X-ray. Nevertheless, we fully acknowledge that symptoms are not often correlated with imaging in COVID-19 infected patients [24].

The most common associated disease in our cohort was the presence of anemia during pregnancy, and pregnancy-induced hypertension. A slightly higher incidence of hypertensive disorder in pregnancy was found in symptomatic pregnant women in our study (12.5%) than in a systematic review published in 2020, where hypertension was present in 8.5% of cases [25].

Analyzing laboratory parameters, a slightly higher frequency of leukocytosis (59.4%) was noted in symptomatic pregnant women, compared to the study of Sinaci et al. [15], in which 27% of pregnant women had leukocytosis [7]. Lymphopenia was also present in a lower percentage (34.3%), compared with pooled data from a systematic review by Juan et al. [26]. Increased CRP values in the group of symptomatic pregnant women were in 87.5% of cases, slightly higher compared with the 75 % in the study of Sinaci et al. [15]. An increased value of CRP was found in 70% of pregnant women in Zaigham and Andersson’s systematic review [27], while in another systematic review by Juan et al. an increased CRP value was recorded in 45.7% of the cases [26].

Increased AST and ALT values were found in 21.8% and 3.12 % of pregnant women, compared with a slightly higher percentage (29.1%) in the study of Sinaci et al. [15]. An increased value of D-dimer was also found in a lower percentage in our study (53.1%) in symptomatic pregnant women, while according to the study of Sinaci et al. increased levels of D-dimer were observed in 91.6% of pregnant women [15]. An increased value of LDH in symptomatic pregnant women was found in 81.2% of pregnant women, which is a higher frequency compared with the meta-analysis by Diriba et al., where it was 34.8% [25].

Antibiotics were used to treat all pregnant women with a SARS-CoV-2 infection, both symptomatic and asymptomatic ones. This was done mainly to prevent superinfection or as prophylaxis before a cesarean section, as was done in other studies summarized elsewhere [27]. Due to their maternal condition, corticosteroids were used in 6.25% of symptomatic pregnant women.

In our cohort analysis, we found a slightly higher—but non-significant—rate of preterm delivery (*p* = 0.673), cesarean section (*p* = 0.185), neonatal low birth weight (*p* = 0.201), and Apgar score ≤ 7 at the first minute (*p* = 0.519). These data are in partial agreement with other studies with similar populations [28,29]. According to recent data, obstetric adverse outcomes might be associated with placental abnormalities, mainly feto-maternal vascular malperfusion [30]; indeed, despite the fact that the angiotensin conversion enzyme 2 (ACE2) expression on placental components was confirmed, there is no agreement on the mother–child vertical transmission of this virus, so the underlying cause is likely to be due to the altered placental vascular framework [31]. As reported in a recent meta-analysis, increased perivillous fibrin deposit; intervillous thrombosis; microscopic accretism; villous edema; increased circulating nucleated red blood cells; or membranes with hemorrhage were the most common abnormal histologic findings in case of maternal COVID-19 infection [32].

Interestingly, in a large systematic review study by Mirbewyk et al. [33], which included 386 pregnant women, there were 257 caesarean sections and 42 vaginal births. The authors hypothesized that the fear of potential vertical transmission of the virus could be, at least in part, one of the reasons for such a high rate of cesarean section. In a National Cohort Survey in the United Kingdom, Knight et al. indicated the percentage of cesarean sections among pregnant women with SARS-CoV-2 infection during pregnancy was 59% [34].

Unlike our data analysis, other authors found that the Apgar score in the first or fifth minute differs significantly in newborns of pregnant women with SARS-CoV-2 infection compared to newborns of pregnant women without infection [9].

Although this can be considered one of the few studies in the setting of Bosnia and Herzegovina that monitored the impact of SARS-CoV-2 infection on obstetric and neonatal outcomes, several limitations should be taken into account for proper data interpretation: first, the retrospective nature of the study could be considered an intrinsic bias; second, the number of enrolled women is relatively low, so the study may be underpowered to detect differences for (at least some of) the parameters we investigated; third, we did not report all the potential comorbidities during pregnancy but just the most common ones; fourth, we did not collect and analyze data about placental macroscopic and microscopic characteristics, nor perform any laboratory investigation on the placental tissue, from COVID-19-positive pregnant women; fifth, all the main parameters were reported as the rate of the event (i.e., pure number and percentages), stratifying continuous variables of subgroups even when it was possible to report means and standard deviations; finally, in this study we analyzed only data from COVID-19 positive women at the moment of hospital admission for delivery, so we could not be sure about when or how long (i.e., at which gestational age) the patient caught the infection. Nevertheless, we did not find (*p* = 0.673) a significant difference for preterm delivery rate; although this may be considered only a surrogate analysis, it allows us to infer that gestational age and symptoms were not correlated in our series.

## 5. Conclusions

In our research setting, symptomatic pregnant women with SARS-CoV-2 infection at the delivery show more frequently altered laboratory parameters compared with asymptomatic ones; moreover, they have a slightly higher but non-significant rate of preterm delivery and of cesarean section, as well as lower neonatal birth weight and Apgar score compared with asymptomatic women.

## Figures and Tables

**Table 1:** Maternal characteristics in pregnant women with SARS-CoV-2 infection.

	Symptomatic, n (%)	Asymptomatic, n (%)	*p*
**Age**			
18–34	27 (84.4)	26 (89.7)	0.71
>35	5 (15.6)	3 (10.3)	
**Parity**			
I	12 (37.5)	14 (48.3)	0.305
II	16 (50)	9 (31)	
>III	4 (12.5)	6 (20.7)	
**Comorbidities in pregnancy**			
Pregnancy-induced hypertension	4 (12.5)	2 (6.9)	0.465
Anemia in pregnancy	7 (21.8)	6 (20.7)	0.912
Hypothyroidism	0	1 (3.44)	0.289
Myomas	1 (3.12)	0	0.337

**Table 2:** Clinical and laboratory data in pregnant women with SARS-CoV-2 infection.

	Symptomatic, n (%)	Asymptomatic, n (%)	*p*
Pneumonia	29 (90.6)	0	0.00001
Leukocytosis; average	19 (59.4); 11.2	5 (17.2); 9.5	0.00078
Lymphopenia; average	11 (34.3); 1.46	1 (3.44); 1.53	0.0024
C reactive protein; average	28 (87.5), 45	15 (51.7); 30.6	0.002
AST; average	7 (21.8); 25.4	0; 24.8	0.007
ALT; average	1 (3.12); 15.1	0; 16	0.377
LDH; average	26 (81.2); 229.5	15 (51.7); 205	0.0142
Ferritin; average	17 (53.1); 35.7	5 (17.2); 39.8	0.0036
D-dimer; average	17 (53.1); 2.8	4 (13.8); 2.58	0.00124
CK; average	3 (9.37)	0	0.091

AST: aspartate aminotransferase; ALT: alanine aminotransferase; LDH: lactate dehydrogenase; and CK: creatine kinase.

**Table 3:** Therapies in pregnant women with SARS-CoV-2 infection.

	Symptomatic, n (%)	Asymptomatic, n (%)	*p*
Antibiotics	2 (6.25)	8 (27.6)	0.024
Antibiotics + LWMH	31 (96.9)	20 (68.9)	0.00328
Corticosteroids	2 (6.25)	0	0.17
Oxygen	5 (16.6)	0	0.026
Admission to the intensive care unit	2 (6.25)	0	0.17

LWMH: low-molecular-weight heparin.

**Table 4:** Maternal and neonatal outcomes in pregnant women with SARS-CoV-2 infection.

	Symptomatic, n (%)	Asymptomatic, n (%)	*p*
Average birth weight			
<2499 gr	5 (15.1)	1 (3.44)	0.201
>2500 gr	28 (84.9)	28 (96.5)	
Time of delivery			
Preterm	4 (12.5)	2 (6.89)	0.673
Term	28 (87.5)	27 (93.1)	
Mode of delivery			
Vaginal	17 (53.1)	21 (72.4)	0.185
Cesarean section	15 (46.9)	8 (27.6)	
Apgar score			
First minute			
≤7	7 (21.2)	4 (13.8)	0.519
≥8	26 (78.8)	25 (86.2)	
Fifth minute			
≤7	1 (3.03)	0	1
≥8	32 (96.9)	29 (100)	

## Data Availability

The full dataset will be available from A.C. on reasonable request.
